# Efficacy of telemedicine-based antimicrobial stewardship program to combat antimicrobial resistance: A systematic review and meta-analysis

**DOI:** 10.1177/1357633X231204919

**Published:** 2023-10-17

**Authors:** Valerie J Dirjayanto, Gilbert Lazarus, Priscilla Geraldine, Nathaniel G Dyson, Stella K Triastari, Jasmine V Anjani, Nayla KP Wisnu, Adrianus J Sugiharta

**Affiliations:** 1 5994 Faculty of Medical Sciences, Newcastle University, Newcastle Upon Tyne NE1 7RU, UK; 2 64733 Faculty of Medicine, Universitas Indonesia, Pondok Cina, Beji, Depok, West Java 16424, Indonesia

**Keywords:** Antimicrobial stewardship, drug resistance, telemedicine, telehealth, systematic review, meta-analysis‌

## Abstract

**Introduction:**

Antimicrobial resistance (AMR) is a major public health threat. Improving antimicrobial use is the main strategy against AMR, but it is challenging to implement especially in low-resource settings. Thus, this review aims to explore the efficacy of telehealth-based antimicrobial stewardship programs (ASP), which is more accessible.

**Methods:**

Registered to PROSPERO and following PRISMA guidelines, literature search was performed in databases including PubMed, Scopus, Cochrane, Science Direct, EBSCOhost, EMBASE, and Google Scholar, searching for studies implementing telehealth ASP. Critical appraisal of studies was performed using Newcastle-Ottawa Scale for Cohort Studies (NOS), Cochrane Risk-of-Bias tool (RoB) 2.0, and Risk Of Bias In Non-randomised Studies-of Interventions (ROBINS-I). We utilized inverse variance, random effects model to obtain the pooled odds ratio (OR) and mean difference (MD) estimates, as well as sensitivity and subgroup analysis.

**Results and Discussion:**

The search yielded 14 studies. Telehealth-based ASP was associated with better adherence to guidelines (pooled OR: 2.78 [95%CI:1.29–5.99], p = 0.009; I2 = 93%), within which streamlining yielded better odds (pooled OR: 30.54 [95%CI:10.42–89.52], p < 0.001) more than the compliance with policy subgroup (pooled OR: 1.60 [95%CI:1.02–2.51], p = 0.04). The odds of antimicrobial prescription rate reduced significantly (pooled OR: 0.60 [95%CI:0.42–0.85], p = 0.005; I2 = 94%), especially for the lower respiratory infection subgroup (pooled OR: 0.37 [95%CI:0.28–0.49], p < 0.001). Days of therapy decreased (pooled MD: -47.12 [95%CI: −85.78– −8.46], p = 0.02; I2 = 100%), with the greatest effect in acute care settings (pooled MD: -97.73 [95%CI:−147.48–47.97], p = 0.0001). Mortality did not change significantly (pooled OR: 1.20 [95%CI:0.69–2.10], p = 0.52; I2 = 63%).

**Conclusion:**

Telehealth-based ASP was proven beneficial to increase adherence to guideline and reduce prescription rates, without significantly affecting patient clinical outcome. After further studies, we recommend more widespread use of telemedicine to combat AMR.

## Introduction

Antimicrobial resistance (AMR) is a leading cause of death globally, currently emerging as the most concerning public health threats of the twenty-first century. It caused more than 1.27 million deaths directly attributable to AMR and 4.95 million deaths associated with AMR in 2019. AMR occurs when antimicrobial drugs are no longer effective enough to kill or inhibit pathogenic organisms, including bacteria, viruses, fungi, and parasites. This causes higher risk of severe infection and subsequently leads to deaths. Many factors such as inappropriate antimicrobial drugs prescriptions, overuse of antimicrobial drugs, and misdiagnosing non-infectious disease as infectious disease are responsible for the rapid progression of AMR.^2,3^

Regulating the use of antimicrobial drugs is the main strategy in our fight against AMR. Antimicrobial stewardship program (ASP) is a global coordinated program in healthcare facilities level that promotes the appropriate prescription of antimicrobial drugs by clinicians. The main target of the program is to improve patient outcomes by preventing severe infection and deaths, decrease antimicrobial resistance, and lower the transmission rate of infections associated with multidrug-resistant organisms.^4^ The Center for Disease Control and Prevention has provided guidelines on strategies to establish successful ASP in clinical settings.^5^ However, currently it is still very challenging to implement a sustainable yet effective ASP in healthcare settings due to insufficient number of trained physicians and pharmacists for implementing ASP, especially in low-resources settings.^6,7^ Moreover, other studies addressed that the root problems may rise from inadequate supervision and poor leadership in each hospital, as well as failure in engaging all relevant stakeholders on critical decision making during ASP.^1^

With the advancement of technology, telemedicine is now used more extensively in many healthcare sectors. Recent studies have shown that using telehealth to deliver ASP might improve its accessibility and efficiency.^8,9^ Telehealth-based ASP utilizes information technology, such as telephones, electronic mails, video conference platforms, or mobile application to facilitate antimicrobial prescription training to healthcare providers in a more inclusive and cost-efficient way than the conventional ASP. The aim is to make ASP more accessible and widely spread, as well as to support the halt of antimicrobial resistance progression.^10^ However, up to the current literature, no study has reviewed the efficiency of telehealth-based ASP from clinical studies. Thus, with this systematic review and meta-analysis, we would like to explore the efficacy of telehealth-based ASP, especially whether it is feasible to be implemented widely in the future, especially since the results from telehealth-based ASP could serve as a breakthrough solution in combating antimicrobial resistance.

## Materials and methods

This systematic review and meta-analysis were conducted according to the Cochrane Handbook for Systematic Reviews of Interventions 6.2 and reported with regards to the Preferred Reporting Items for Systematic Review and Meta-Analysis (PRISMA). This study has also been registered to the International Prospective Register of Systematic Review (PROSPERO).

### Information sources and search strategy

Literature search was performed in databases including PubMed, Scopus, Cochrane, Science Direct, EBSCOhost, EMBASE, and Google Scholar, searching for studies implementing telehealth antimicrobial stewardship programs (ASP) from inception up May 2023 with keywords detailed in Table 1.

**Table 1.** Search databases and keywords.

**Database**	Keywords
PubMed	(“Telemedicine”[Mesh] OR “Remote Consultation”[Mesh] OR TeleHealth[Text Word] OR eHealth[Text Word]) AND (“Drug Resistance, Microbial”[Mesh] OR “Antimicrobial Stewardship”[Mesh] OR (antibiotic AND resistance))
ScienceDirect	(Telemedicine) AND (Drug OR Antimicrobial OR antibiotic) AND (resistance OR stewardship)
Google Scholar	(Telemedicine) AND (Antimicrobial OR antibiotic) AND (resistance OR stewardship)
EBSCOHost (MEDLINE)	(Telemedicine) AND (Drug OR Antimicrobial OR antibiotic) AND (resistance OR stewardship)
Embase	(‘telemedicine'/exp OR telemedicine OR ‘telehealth'/exp OR telehealth OR ‘telecare'/exp OR telecare) AND (‘drug'/exp OR drug OR ‘antimicrobial'/exp OR antimicrobial OR ‘antibiotic'/exp OR antibiotic) AND (‘resistance'/exp OR resistance OR stewardship)
Scopus	(“Telemedicine”) AND (“Drug” OR “Antimicrobial” OR “antibiotic”) AND (“resistance” OR “stewardship”)
Cochrane	MeSH descriptor: [Telemedicine] explode all trees AND (MeSH descriptor: [Drug Resistance, Microbial] explode all trees OR MeSH descriptor: [Antimicrobial Stewardship] explode all trees OR (antibiotic) OR (stewardship))

### Study eligibility criteria

Along with study screening, the authors predetermined the following inclusion criteria: (1) type of study, clinical studies including cohort, cross-sectional studies, and clinical trials; (2) study population, clinicians, and patients from any healthcare settings level; (3) intervention, telehealth-based antimicrobial stewardship programs; (4) outcomes, including adherence to guidelines, antimicrobial prescription, days of therapy, and mortality rate.

Definitions of outcomes were determined as follows: adherence to guidelines was the extent of conformity between perception, knowledge, and action with guideline recommendations. Antimicrobial prescription was the amount of antibiotics given for any particular infectious disease. Days of therapy (DOT) or antimicrobial days were the duration of administration of antibiotics as typically documented in the medical records. Meanwhile, mortality rate was the frequency of death within a specific subject in a certain period.

Meanwhile, the exclusion criteria were set to: (1) unsuitable study design, including non-clinical trials; (2) studies with incomplete outcome data; (3) studies which were not completed yet at the time of retrieval; (4) studies with irretrievable full-text articles; and (5) studies without a control group.

### Data extraction

We predetermined the outcome sheet in tabular form (MS Excel MS Excel® for Mac; Microsoft Corporation, Redmond, WA, 2018) to include the following data to be extracted: (1) author and year of publication; (2) study characteristics, including study design and location of study; (3) study population, including sample size, mean age, and the healthcare facilities’ characteristics; (4) intervention, type of telehealth modalities used to present ASP training and duration of intervention; and (5) study outcomes, including the assessed parameters, values with and without intervention, as well as significance (p-values). Qualitative characteristics were extracted by six reviewers, and two independent authors rechecked accuracy of extracted data while performing statistical analysis.

### Quality assessment

Quality of each study was assessed by six independent reviewers and should there be any disagreement, resolution would be made based on consensus from the six reviewers. Cohort studies were assessed using Newcastle-Ottawa Scale designed for Cohort Studies (NOS) and converted into the Agency of Healthcare Research Quality (AHRQ) standards into good, fair, or poor quality. This tool consists of quality and risk of bias assessment based on the broad perspective including study selection, comparability of the groups, and the ascertainment of the exposure of interest.

On the other hand, for other non-cohort studies, we utilized the Risk of Bias in Non-Randomized Studies of Interventions (ROBINS-I) tool of bias assessment. The ROBINS-I tool consists of seven main domains. The first two domains covering confounding and selection of participants into the study address issues before starting the interventions that are to be compared. The third domain addresses classification of the interventions themselves. The other four domains address issues after the start of interventions: biases due to deviations from intended interventions, missing data, measurement of outcomes, and selection of the reported results.

Randomized controlled trials were assessed using the Cochrane Risk-of-Bias (RoB) 2.0. The Cochrane RoB 2.0 tool consist of 5 domains, including the risk of bias from randomization process, deviations from intended interventions, missing data, measurement of outcomes, as well as selection of the reported results.

### Statistical analysis

We performed statistical analysis using Review Manager ver. 5.4 (The Nordic Cochrane Center, The Cochrane Collaboration, Copenhagen). The odds ratio with 95% confidence interval (CI) and p-value were extracted from studies and we interpreted the pooled effects. For analyzing two parameters, namely mortality and guideline adherence, we utilized odds ratio (OR) data with a 95% confidence interval and its respective p-value. If OR >1, telehealth ASP was associated with lower mortality rate or better guideline adherence, thus, means that telehealth ASP is beneficial compared to standard ASP. Factors were put as study code, log of odds ratio, and standard of error which will be calculated for study weight, fixed odds ratio, and also its 95% confidence interval (CI) which will be presented in forest plot. Moreover, for assessing days of therapy and length of stay, we utilized mean differences (MD) and standard deviations (SDs) and we interpreted the results. We utilized an inverse variance, random effects model, since we considered that individual-specific effects of the factors associated will be contrasted and highlighted more.^11^ Heterogeneity was further evaluated using I2 statistics, with cut-off limits of 0%, 25%, 50%, and 75% as insignificant, low, moderate, and high heterogeneity, respectively.^12^ Additionally, we performed sensitivity analysis following the Duval and Tweedie's trim-and-fill method to identify any outlier study, followed by subgroup analysis based on the factors that might contribute to the heterogeneity.

## Results

### Search results and study selection

**Figure 1.**
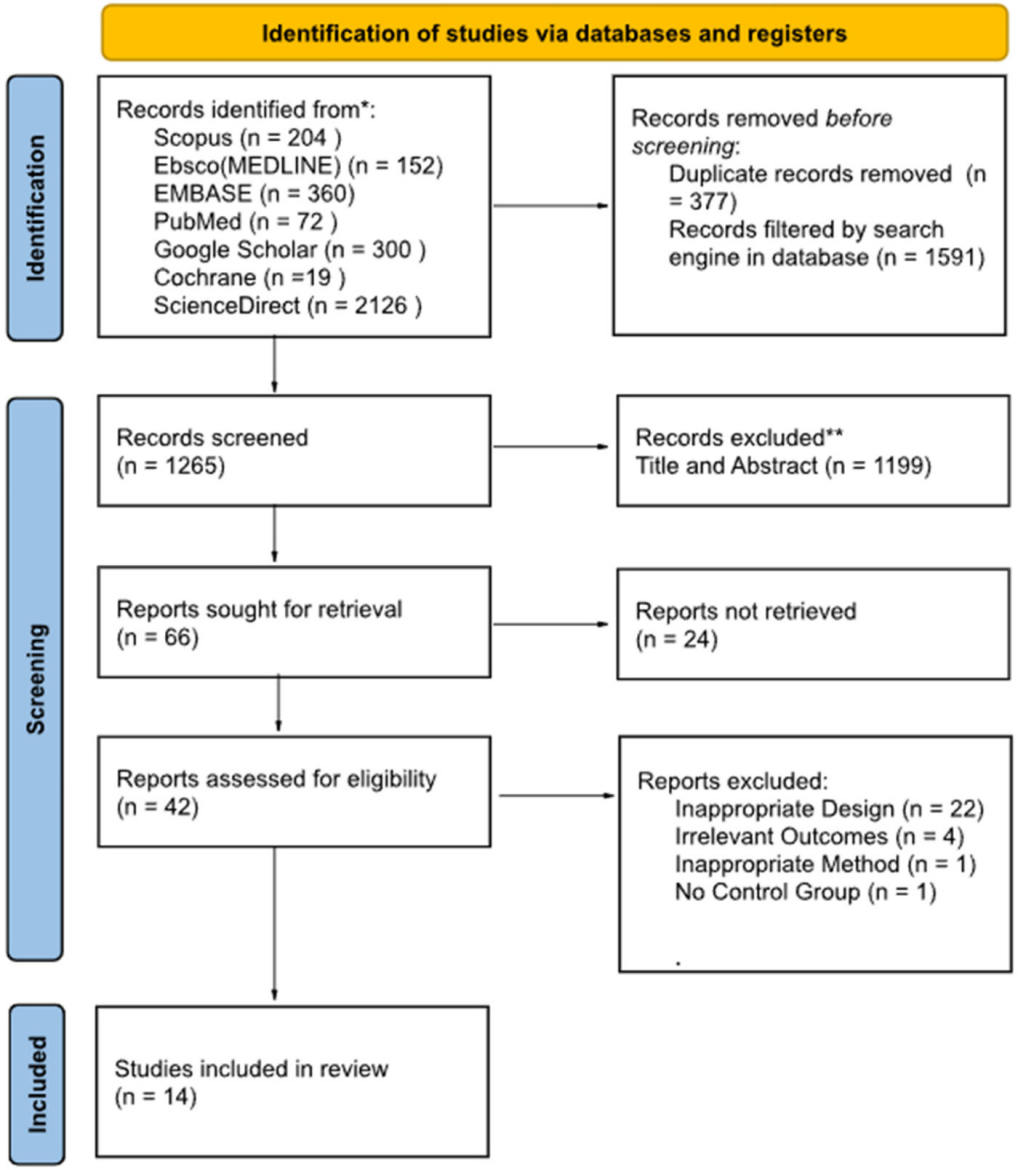
PRISMA flow diagram of literature search strategy.

Upon database searching, 3233 records were identified, after which 377 duplicates were removed, and 1591 articles marked as ineligible by automation tools. Primary filtering via title and abstract screening removed 1199 articles, with 42 articles finally assessed for full-text eligibility. On the whole, we found 22 studies with unsuitable design, 4 with irrelevant outcome, 1 with inappropriate method, and 1 without any control group. Finally, 14 studies were included and systematically reviewed in this study. Screening of titles and abstracts of studies was carried out by six independent reviewers. The planned procedure is illustrated in Figure 1.

### Study characteristics and design

All 14 studies included are trials in clinical settings, consisting of 5 retrospective cohort studies, 2 quasi-experimental studies, 3 clinical trials, 2 observational longitudinal studies, and 2 comparative (pre-post) studies. These studies were conducted in several regions including 10 studies in the United States, 1 study in Brazil, 1 study in the United Kingdom, 1 study in Australia, and 1 study in Italy. Among the included studies, telehealth antimicrobial stewardship programs are delivered either by telephone, electronic mail, video conference, or mobile application within the study period. Outcomes are measured via parameters including antibiotic prescriptions, mean length of stay, 30-day mortality, or adherence to guidelines. The complete characteristics of included studies are shown in Online Appendix 1.

### Results from quality assessment

**Figure 2.**
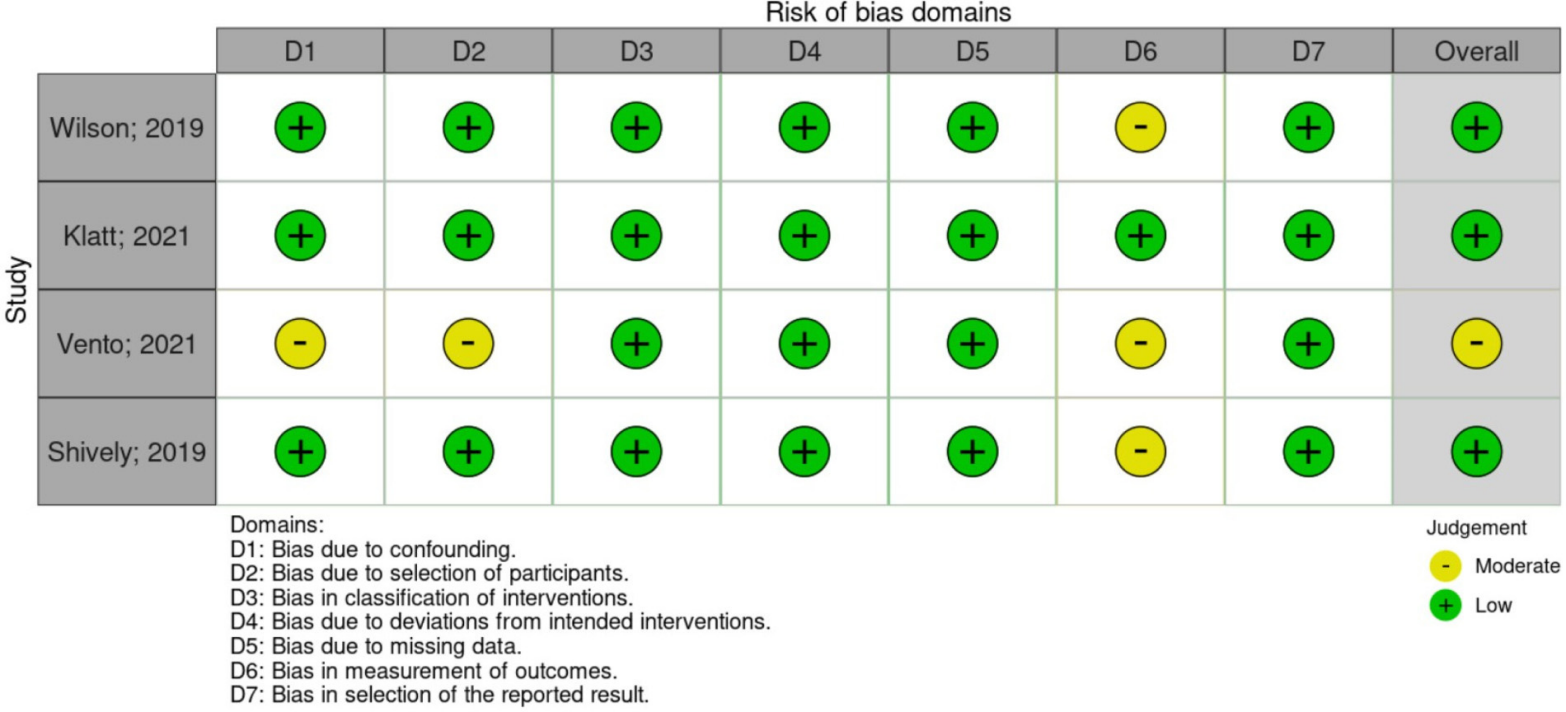
Risk of bias assessment for intervention studies using ROBINS-I.

**Figure 3.**
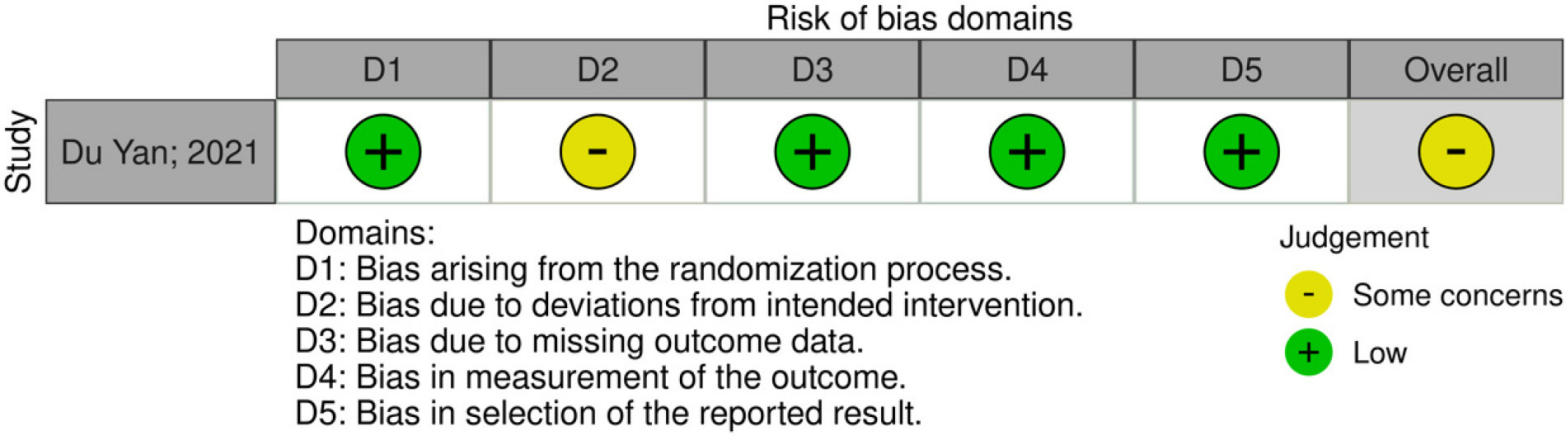
Risk of bias assessment for cohort studies using cochrane ROB 2.0.

**Figure 4.**
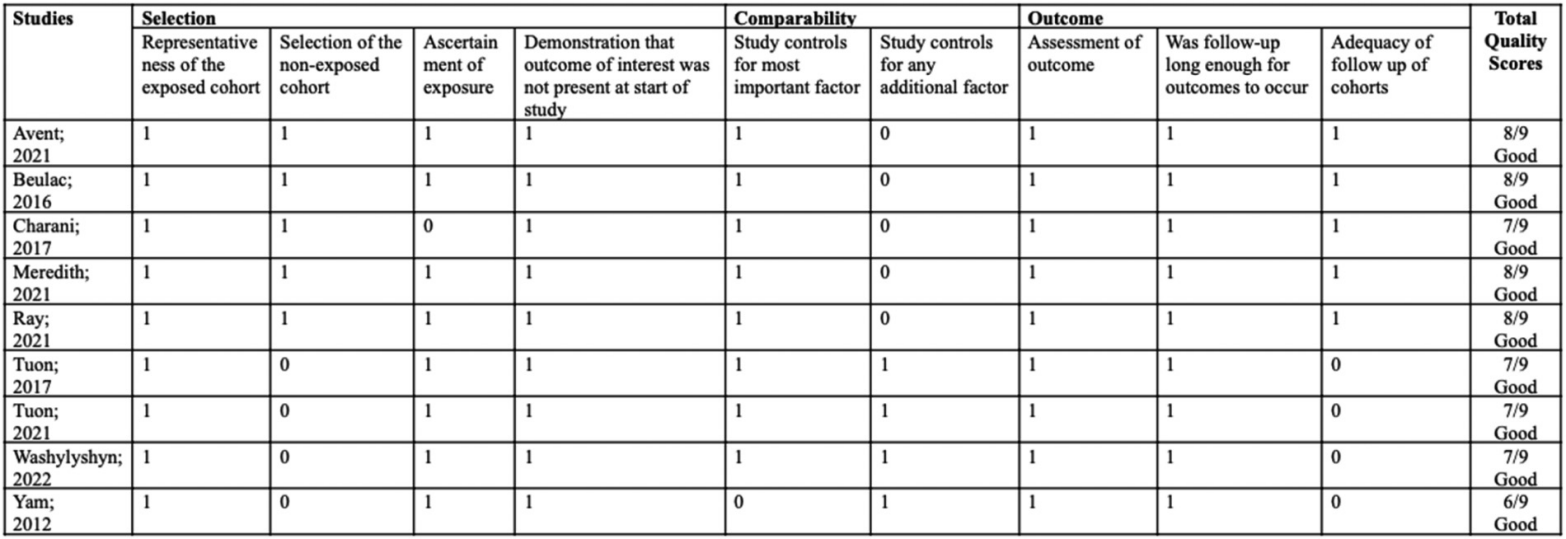
Risk of bias assessment for cohort studies using NOS.

Risk of bias assessment was conducted with the results as shown in Figure 2 from the ROBINS-I tool, Figure 3 from the Cochrane RoB 2.0, and Figure 4 from the NOS-cohort criteria. The detailed bias assessment is presented in Online Appendix 2 and 3, and funnel plots are presented in Online Appendix 4From the ROBINS-I tool, we could draw a conclusion that most studies show good quality, except for some concerns in Vento et al.'s study, particularly in the first, second, and sixth domains. Furthermore, from the NOS-cohort tools, we discovered that all studies have a low risk of bias, except for Yam et al.'s study. The bias detected from this study was caused by an absence of selection from the non-exposed cohort, the lack of study control for the most important factor, and inadequacy of follow up of cohorts. However, both studies have no bias regarding outcome assessment and reporting results. Du Yan et al.'s study with Cochrane RoB 2.0 showed some concerns in blinding due to the nature of the intervention, which was unavoidable. Overall, these may not interfere considerably with the general quality of our meta-analysis.

### Telemedicine methods

In AMS, telemedicine implemented involves attempts in improving antimicrobial utilization through technological advancements typically involving feedback, assessment, or audit from a remote infectious medicine consultant or guidelines, which increases accessibility in conditions where these infectious medicine specialists are less reachable. The studies included in this review used different methods of conducting telemedicine. For example, Vento et al., Shively et al., and Avent et al. used telephone calls as the way to conduct telemedicine education intervention. On the other hand, some studies used mobile applications, such as those from Charani et al., Klatt et al., and Tuon et al. Another different method was also shown by Wilson et al., Meredith et al., Ray et al., and Yam et al., in which they utilized video conferences to convey the education on antimicrobial stewardship, whereas Beulac et al. used email to conduct their intervention. Similar technique was also used in Du Yan et al.'s study, in which they combined an online education on antimicrobial stewardship and an online dashboard which provided individualized feedback to patients related to their antimicrobial uses. Wasylyshyn et al. modified an online questionnaire to support antimicrobial stewardship in an established online portal which provided telemedicine and could be accessed by the patients prior to hospital visit. Lastly, Ceradini et al. developed a software in which many physicians and specialists were able to conduct biweekly online meetings to discuss the treatment for their patients and provide antimicrobial guidance for each of their own patients.

### Adherence to guideline

**Figure 5.**
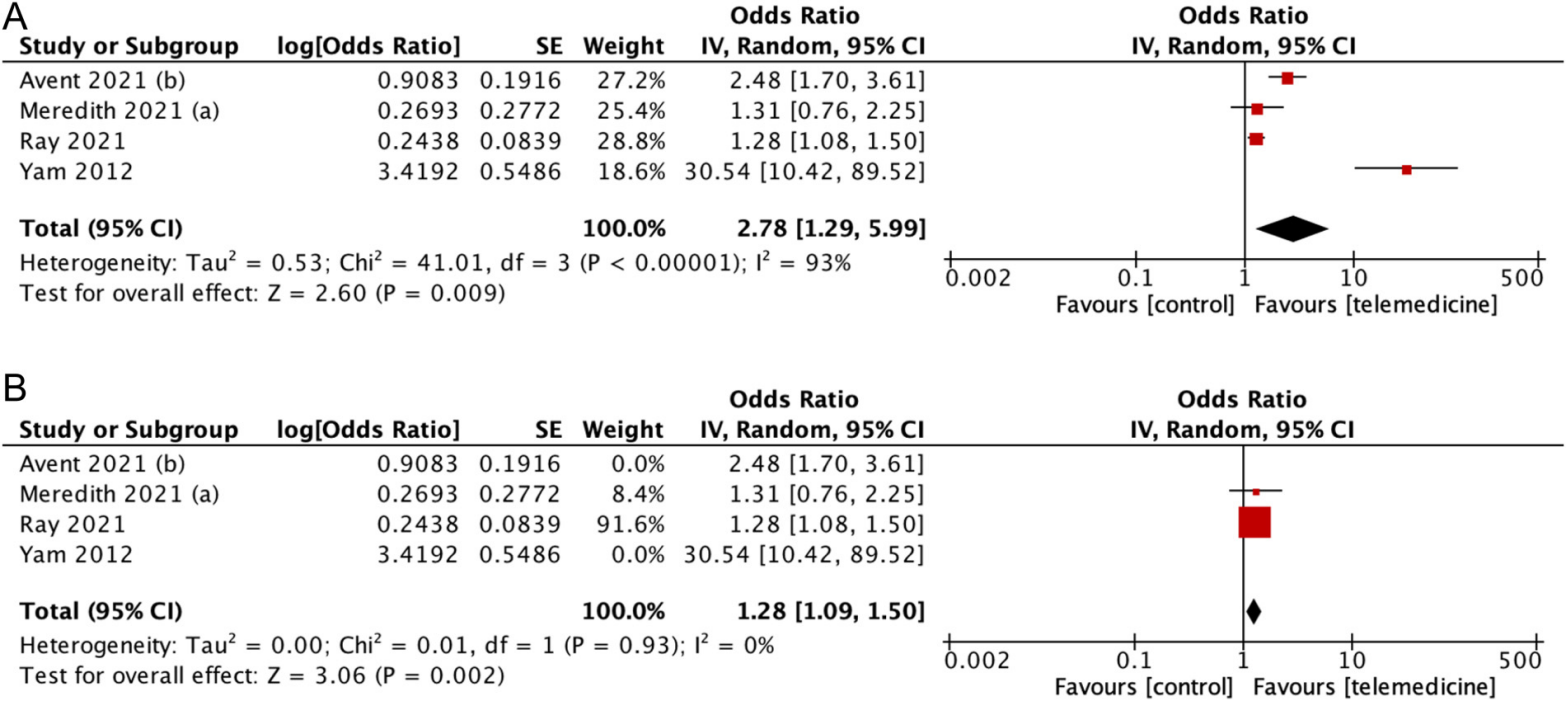
(A) Forest plot showing effect of telemedicine on adherence to guideline (B) sensitivity analysis for studies assessing telemedicine effect to adherence to guideline.

In this meta-analysis, the combined odds ratio [pooled OR = 2.78 (CI95%: 1.29,5.99); p = 0.009; I2 = 93%] showed that there was an inclination to a higher rate of adherence using telemedicine strategies, though with a considerable amount of heterogeneity (I2 = 93%) (Figure 5). Further sensitivity analysis identified Avent et al. and Yam et al. as outliers, with their removal reducing heterogeneity to 0% (pooled MD = 1.28 [95%CI: 1.09,1.50, p = 0.002, I2 = 0%]).

**Figure 6.**
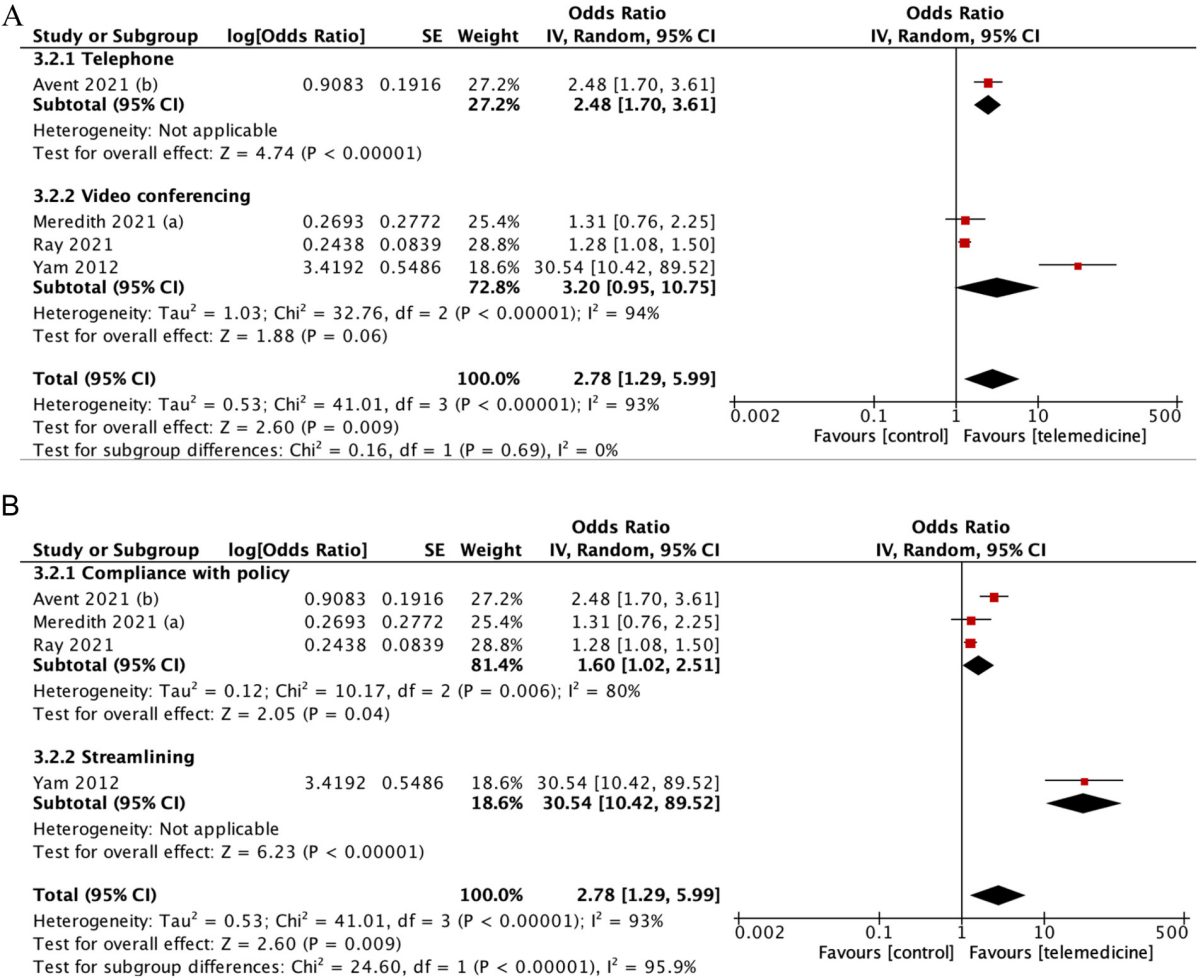
(A) Subgroup analysis according to the media of telemedicine used (B) subgroup analysis according to aspects of adherence used as the definition.

Subgroup analysis indicated that the use of video conferencing showed a stronger effect (pooled OR: 3.20 [95%CI: 0.95–10.75], p = 0.06) compared to the telephone subgroup (pooled OR: 2.48 [95%CI: 1.70–3.61], p < 0.001), although the differences were not statistically significant (P = 0.69) (Figure 6). Furthermore, the effect was found to be more profound with streamlining (pooled OR = 30.54 [95%CI: 10.42–89.52]; p < 0.00001] compared to compliance with policy (pooled OR = 1.60 [CI95%: 1.02–2.51]; p = 0.04; I2 = 80%].

### Antibiotic prescription

**Figure 7.**
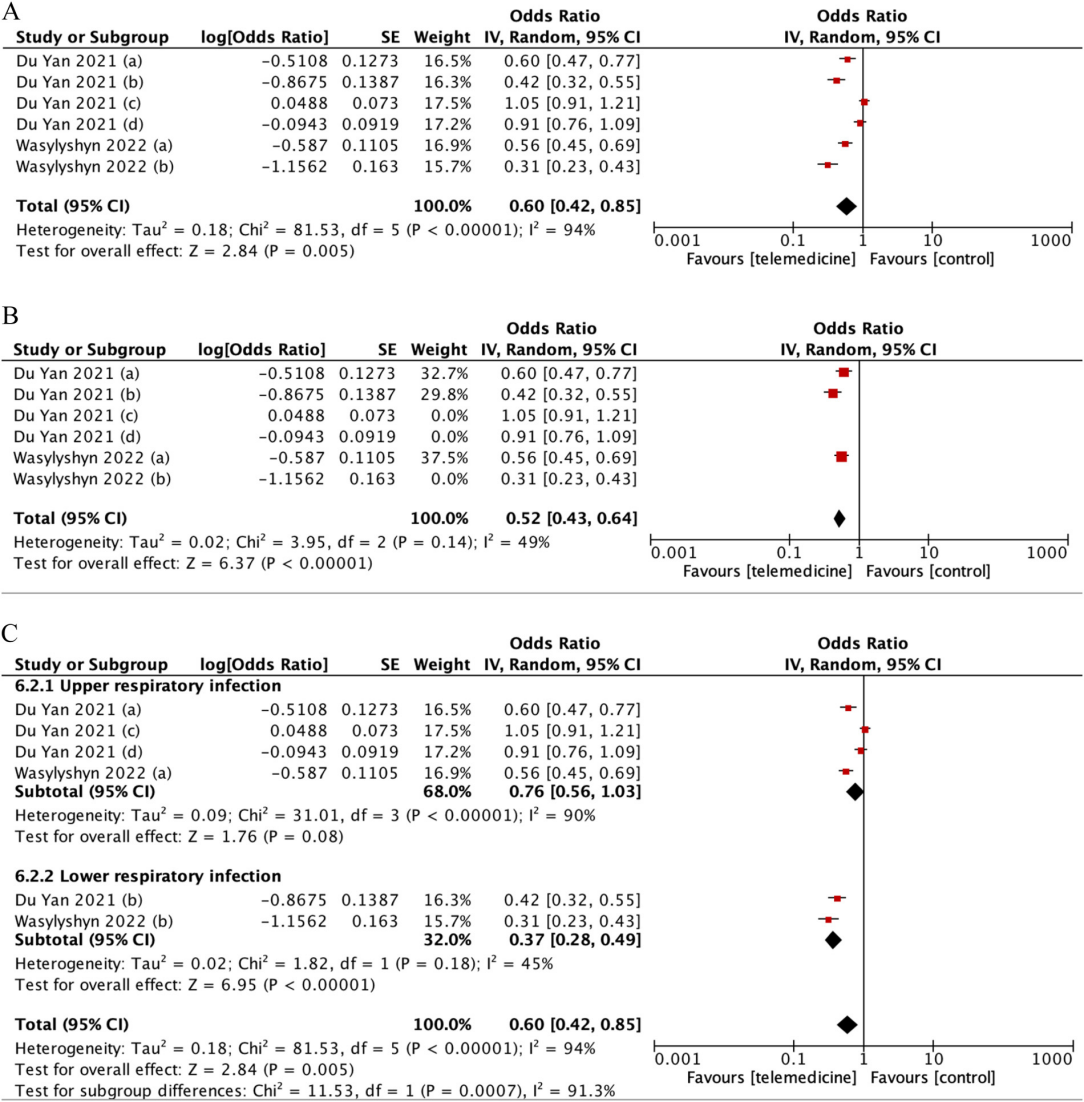
(A) Forest plot showing effect of telemedicine on antimicrobial prescription. (B) Sensitivity analysis for studies assessing telemedicine effect to antimicrobial prescription. (C) Subgroup analysis according to upper-lower respiratory infection.

The combined odds of prescribing antimicrobials was significantly lower with telemedicine strategies, despite high heterogeneity (pooled OR: 0.60 [95%CI: 0.42–0.85], p = 0.005; I2 = 94%) (Figure 7). Further identified in the subgroup analysis, the odds of antimicrobial prescription was significantly reduced in lower-respiratory infection (OR: 0.37 [95%CI:0.28–0.49], p < 0.001) when compared to upper-respiratory infection (OR: 0.76 [95%CI:0.56–1.03], p < 0.001).

### Days of therapy (DOTs)

**Figure 8.**
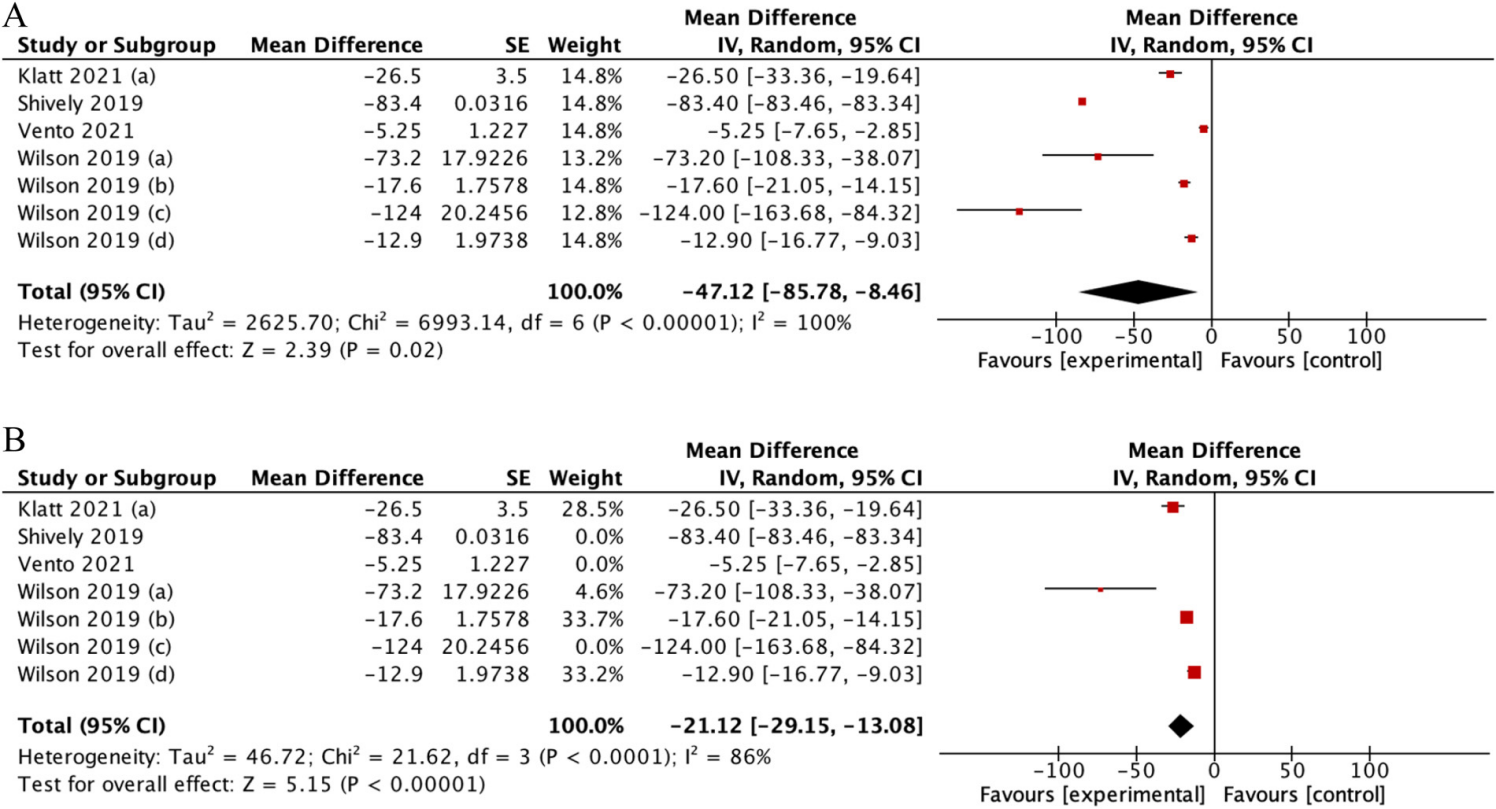
(A) Forest plot showing effect of telemedicine on DOTs. (B) Sensitivity analysis for studies assessing telemedicine effect of telemedicine on DOTs.

Details of statistical calculations for days of therapy are available in Online Appendix 5. Our analysis demonstrated that tele-stewardship decreased days of therapy, yielding a pooled mean difference (MD) of −47.12 (95%CI: −85.78–8.46) (Figure 8). However, high heterogeneity was found (I2 = 100%), with it decreasing to I2 = 86% upon sensitivity analysis removing Shively et al., Vento et al., and Wilson et al.'s studies as outliers.

**Figure 9.**
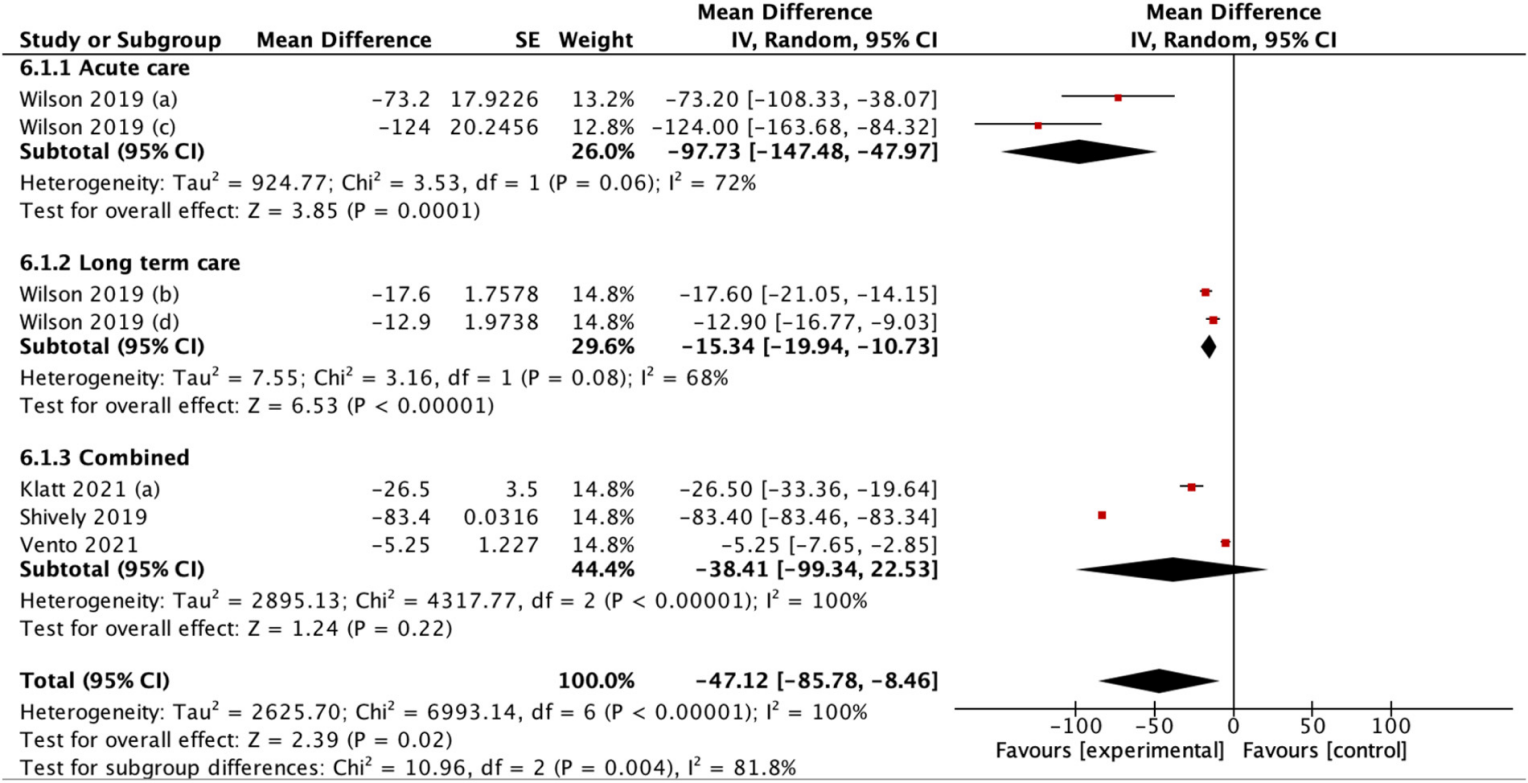
Subgroup analysis according to acute or long-term care.

Further identified in the subgroup analysis, days of therapy significantly decreased in long-term care use (pooled MD −15.34 [95% CI: −147.48– −47.97], p = 0.0001; I2 = 72%) when compared to use in acute care (pooled MD −97.73 [95% CI: −19.94– −10.73], p < 0.00001; I2 = 68%) or both combined (pooled MD −38.41 [95% CI: −99.34– −22.53], p = 0.22, I2 = 100%) (Figure 9). This difference was statistically significant (p = 0.02), suggesting that tele-stewardship yielded most effective application in long-term care.

### Mortality rate

**Figure 10.**
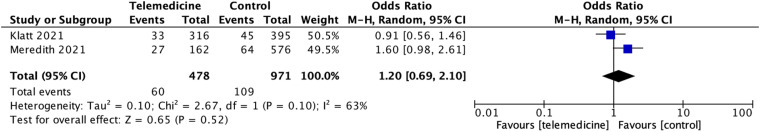
Forest plot showing effect of telemedicine on mortality rate.

Results from the meta analysis indicated that mortality rate did not improve with tele- stewardship, with a pooled MD of 1.20 (95% Cl:0.69 = 2.10, p = 0.52). The results also displayed substantial heterogeneity, with I2 of 63% (Figure 10).

## Discussion

### Adherence to guideline

In clinical practice, it is essential to encourage guideline adherence as it can improve patient outcomes by implementing evidence-based physician practices.^13,14^ In AMS program, guidelines recommended by organizations such as Infectious Diseases Society of America (IDSA) and the Society for Healthcare Epidemiology of America (SHEA) have been published to help improve the use of antimicrobials which is one among several measures required to prevent antimicrobial resistance.^15,16^

The combined odds ratio showed that there was an inclination towards a higher rate of adherence using telemedicine strategies. This could be due to an increased access to consultation with infectious disease physicians or pharmacists which can be unavailable in rural settings.^10^ Considerable amount of heterogeneity was detected, but further sensitivity analysis shows that decreasing heterogeneity to 0% was achieved by excluding studies by Avent et al. and Yam et al.^15,17^ These studies were conducted in rural settings, which might explain the greater effects and variability during impalementation.

The use of video conferencing showed a stronger effect compared to the use of telephone, suggesting that video conferences could be considered more for telehealth-based ASP. In addition, instead of simply compliance with policy, Yam et al.'s study assessed streamlining as part of guideline adherence, which gained more profound effect due to prescribers gaining more confidence to provide suitable AMS recommendations and work autonomously with the presence of telemedicine.^15^

### Antimicrobial prescription

Prudent prescribing and the utilization of antimicrobials in the most appropriate goal-directed strategy to treat or prevent human infectious diseases are goals aspired to be reached by healthcare professionals. Various institutions including the National Health Service (NHS) in United Kingdom have established prescription and stewardship guidelines and policies to reduce number of inappropriate prescriptions and avoid the emergence of resistant microbes.^18^

As seen in the results of this meta-analysis, the odds of prescribing antimicrobials was significantly lower when telemedicine strategies were applied. This reduction might be attributed to education and stewardship feedback. If clinicians’ performance had been continuously evaluated, the number of unneeded prescriptions would be impacted. Wasylysyn's study proposed that physician-champion feedback from stewardship teams were responsible for the change in prescription rates, and this outcome was supported by other literatures from Huang et al. and Camins et al*.*^19–21^ It is important to note that results on this topic still varied when telemedicine was compared with real-time consultations. High heterogeneity was found, which can be attributed to the variation of telemedicine strategies and infections involved in the different studies.

To account for this heterogeneity, we further identified subgroups to analyse more specifically the effects of intervention when implemented on different infections. The odds of antimicrobial prescription were significantly reduced in lower-respiratory infection when compared to upper-respiratory infections, which would yield great implications as lower-respiratory infection typically required more antibiotics. In fact, in contrast to viral upper-respiratory infections which are usually not treated with antibiotics, regardless of etiology, antimicrobials are usually prescribed for pneumonia.^22^

Appropriate antimicrobial dosing and prescription are emerging concerns globally. For instance, in Nigeria, irrational prescription is a major concern, with only 23% out of all prescribed patients confirmed having actual bacterial infection.^23^ In addition, many prescriptions are written empirically.^24^ Stewardship programmes act as a reminder on patient management guidelines, and telemedical stewardship program benefits from lower costs and better access for suburban hospitals/facilities, increasing accessibility, thus capable of reaching larger scope of clinicians.^25^ Hence, rate of antimicrobial prescription is a proper hallmark of a successful stewardship.

### Days of therapy (DOT)

Baseline measurement for antimicrobial utilization is needed to evaluate antimicrobial stewardship initiatives.^26,27^ Studies included in this meta-analysis favored the experimental group for DOT. This decrease in DOT may also imply reduction of associated adverse events, which are common in hospitalized patients, mostly linked to unnecessary antibiotic prescriptions.^28^ However, the heterogeneity of these results was high, which can be attributed to the various scenarios in which antibiotics were used in these studies. Thus, its use in acute and long-term care settings, as well as both combined, were identified as subgroups in our analysis. The use of tele-stewardship had the greatest effect in long-term care. This may be due to the higher infection risk that long-term care patients experience, which might contribute to the overuse of antibiotics in the first place, pre-telestewardship.^29^

Wilson et al.'s study suggested that telestewardship could result in alteration in antibiotic usage due to more robust and flexible communication with infectious disease experts in small and rural healthcare facilities.^30^ This minimized redundant antibiotic prescriptions and feedback between local physicians and experts, allowing an effective usage of antibiotics.^26,30^

### Mortality

In studies involved in this review, to determine an expected mortality rate for each hospital, data obtained from discharge coding was utilized. This involved employing a statistical model to predict the number of deaths that a hospital would typically experience, taking into account the characteristics of the patients admitted.^28^ Result from this analysis showed that mortality rate did not improve with intervention, and heterogeneity was substantial. The reason for this might be that although telehealth-based stewardship could reduce inappropriate prescriptions while enabling real-time monitoring of patient health, it may not be suitable in all cases during management.^29^ Traditional in-person advise from specialists may still be essential for clinical decisions in urgent and complicated cases. Hence, while telemedicine could aid in guiding antimicrobial prescriptions, there was insufficient evidence to suggest that it significantly reduced mortality rates among patients.^30,31^ Furthermore, since only two studies reported mortality as an outcome, further studies are encouraged to include this important clinical measure in order to better evaluate effectiveness.

### Other outcomes

Our review also considered other outcomes, although quantitative analysis was not performed due to data limitations. Klatt et al.^9^ discovered significant reduction in the mean length of stay of patients, suggesting that there might be more rapid improvements in clinical outcomes of patients. Tuon et al.^35^ assessed the bacterial sensitivity directly from the culture, demonstrating that the culture sensitivity to meropenem, polymyxin, and cefepime increased through the usage of telemedicine. This shows that telemedicine intervention may decrease antimicrobial resistance directly. Another outcome is the cost saving effect. According to Tuon et al., the net saving was almost USD 300,000/ year.^35^ Shively et al. also assessed the expenditure saved, which was $142,629.83 annually.^36^

### Clinical implications

Antibiotics stewardship programs are important to counter the growing antimicrobial resistance problem. The core goal of antibiotic stewardship is better care through appropriate use of antibiotics, including appropriate indication, selection of agent, dose, and duration of therapy.^37,38^ This does not always mean using less antibiotics, although it was commonly seen in our studies.^8,9,36^ Although no significant improvement of clinical outcomes was observed overall, patients receiving less antimicrobials might also benefit from reduced likelihood of multiple adverse drug effects.^41^ Our study highlighted that in terms of adherence to guideline, reducing prescription rates, and decreasing days of therapy (DOT), telehealth-based ASP was effective and therefore can be considered for routine use. Similarly, across different disciplines, a previous review of meta-analyses suggested that telehealth in various modalities was effective.^42^ It is also important to highlight that this use of telehealth would be beneficial only if it does not yield unsafe or inferior outcomes compared to standard care, as supported by Snoswell et al. who reviewed 24 meta-analyses and concluded that telehealth did not cause detrimental effects in terms of mortality.^43^

The use of telemedicine in implementing antibiotics stewardship programs could also aid in reaching smaller, more rural hospitals. Smaller hospitals face more barriers in implementing antibiotics prescription guidelines, such as lack of infectious diseases-trained staff and limited microbial laboratory resources.^8^ Telemedicine allowed multidisciplinary teams of professionals to communicate, discuss cases in real time, and establish consistent communication. Most antibiotic stewardship teams consisted of at least an infectious disease-trained physician and pharmacist. The use of videoconference may stimulate face-to-face meetings from individuals in different locations, allowing more flexible meetings between clinicians and antibiotic stewardship team.^30^ The use of asynchronous applications or email could also be beneficial, and even more effective. Study by Beaulac et al. shows that ASP given through email took approximately 1–2 h per week to do for infectious disease specialists.^37^ Vento et al. also compared the time taken for telephone consultation vs asynchronous electronic consultation and found that asynchronous consultation needed significantly less time.^8^ The use of applications in Tuon et al.'s^35^ and Charani et al.'s^38^ study even did not involve any multidisciplinary team, relying on available guidelines and policy. This showed that telemedicine minimized the number of specialists needed to maintain ASP, which might be beneficial in areas with limited infectious disease specialist resources.

This intervention would not be beneficial if it is not applicable in clinical settings. There are at least 3 main reasons why this intervention is applicable to solve current problems in limited-resource settings. First, the use of telemedicine allows consultation and recommendation regarding antibiotics prescription to be given remotely. This allows sustainable education, surveillance, and support towards clinicians from antibiotics stewardship teams, regardless of geographical constraints. This is especially applicable in archipelagos with remote hospitals, which may have less resources than larger hospitals and are less likely to meet the core elements of antibiotics stewardship. Second, the use of technology has significantly risen as of late, especially due to the COVID-19 pandemic, allowing this innovation to be more accepted by the community. Third, the use of telemedicine has the potential to save up cost and energy, from both clinicians and antibiotics stewardship team, therefore suitable with the financial background of developing countries.

## Strength and limitations

This study was the first systematic review and meta-analysis exploring the use of telemedicine in antibiotics stewardship programs. This study included several different kinds of telemedicine, such as telephone, e-mail, video conference, and applications, in the hopes that it might be generalized across all forms of telemedicine. Furthermore, we also included studies involving small rural hospitals, therefore highlighting the use of telemedicine in connecting individuals despite their geographical challenges in solving the problem of antibiotics overuse.

However, this study was not without limitations. There was considerable heterogeneity in some of the outcome measures. Most studies included erre in developed countries, with only one study located in a developing country. Characteristics of the study population and differences in available technology may limit the applicability of this intervention, especially in healthcare facilities lacking similar resources. Moreover, there were only two studies which report mortality as an outcome, and thus further studies are encouraged to incorporate more clinical measures in addition to prescription rate changes.

## Conclusion

Antimicrobial resistance is a major concern worldwide. Appropriate antimicrobial use is crucial to reduce antimicrobial resistance and to further improve patient outcomes. Based on this review, telemedicine antimicrobial stewardship proves to be beneficial to increase adherence to guideline, reduce prescription rates, and decrease days of therapy (DOT), without significantly affecting mortality rate. Also, telemedicine seems to have a better outcome on lower-respiratory infection in terms of improving antimicrobial prescription. Moreover, DOTs benefit more from the usage of telemedicine in acute care.

Given its benefits and applicability, through this comprehensive review, we recommend further studies, after which telemedicine can be utilized more extensively to improve antimicrobial usage. We hope that strategies to telemedicine in antimicrobial usage may be implemented on a large scale based on this comprehensive assessment. This knowledge may also raise professional and governmental attention to implement creative strategies of appropriate antimicrobial usage and promote awareness on the importance of appropriate antimicrobial usage. Furthermore, we hope that appropriate antimicrobial usage may be promoted, for better outcomes for the patient, and less antimicrobial resistance.

## Supplemental Material

sj-docx-1-jtt-10.1177_1357633X231204919 - Supplemental material for Efficacy of telemedicine-based antimicrobial stewardship program to combat antimicrobial resistance: A systematic review and meta-analysis Supplemental material, sj-docx-1-jtt-10.1177_1357633X231204919 for Efficacy of telemedicine-based antimicrobial stewardship program to combat antimicrobial resistance: A systematic review and meta-analysis by Valerie J Dirjayanto, Gilbert Lazarus, Priscilla Geraldine, Nathaniel G Dyson, Stella K Triastari, Jasmine V Anjani, Nayla KP Wisnu, and Adrianus J Sugiharta in Journal of Telemedicine and Telecare
