# Supplementary material for: Efficacy of telemedicine-based antimicrobial stewardship program to combat antimicrobial resistance: A systematic review and meta-analysis
Source: J Telemed Telecare. 2023 Oct 17;31(5):615–27. doi: 10.1177/1357633X231204919 (PMC12095883; doi:10.1177/1357633X231204919)

**Appendices**

**Appendix 1. Details of study characteristics and outcomes.**

| **Author; year** | **Study design** | **Location** | **Population** | | | **Intervention** | **Duration of intervention** | **Period of study** | **Outcomes** | | | |  |
| --- | --- | --- | --- | --- | --- | --- | --- | --- | --- | --- | --- | --- | --- |
|  |  |  | Hospital and prescription number | Patient characteristics | Mean age |  |  |  | Parameter | Value with intervention (SD) | Value without intervention  (SD) | P value | Notes |
| Vento^8^; 2021 | Non-randomized trial | USA | 16 small community hospitals in the Intermountain Healthcare system (n=2,487) | NR | 63 | Advice line interactions via phone calls (n=859),  eConsults (n=761)  telemedicine consultations (n=867) | 18 months | May 2015-April 2018 | Meropenem use (DOT/1,000 DP) | 9 | 13 | 0.03 |  |
|  |  |  |  |  |  |  |  |  | Vancomycin use (DOT/1,000 DP) | 75 | 53 | <0.01 |  |
|  |  |  |  |  |  |  |  |  | Fluoroquinolone use (DOT/1,000 DP) | 82 | 147 | <0.01 |  |
|  |  |  |  |  |  |  |  |  | Piperacillin-tazobactam use (DOT/1,000 DP) | 85 | 59 | 0.09 |  |
| Tuon^32^; 2017 | Observational longitudinal | Brazil | In a university hospital with 186 beds, 37 in ICU (n=922) | NR | NR | Antimicrobial guide in a mobile application consisting of a guidance manual | 12 months | January 2014-December 2015 | Culture sensitivity to meropenem (%) | 83 | 73 | <0.05 |  |
|  |  |  |  |  |  |  |  |  | Culture sensitivity to polymyxin (%) | 83 | 69 | <0.05 |  |
|  |  |  |  |  |  |  |  |  | Culture sensitivity to cefepime (%) | 57 | 62 | <0.05 |  |
|  |  |  |  |  |  |  |  |  | net saving (USD) | 296,485.90 |  | <0.05 |  |
| Shively^33^; 2019 | Non-randomized clinical trial | USA | 2 community hospitals with 285 and 176 beds  (n=1,419) | Patients admitted with lower respiratory tract infections or skin and soft tissue infections, with broad-spectrum antibiotic use | NR | Telehealth-based antimicrobial stewardship remotely made by infectious disease physicians to primary teams via telephone calls for 60 minutes, 3 times weekly at a 285-bed hospital and 2 times weekly at a 176-bed hospital | 6 months | March 2017-August 2018 | Broad-spectrum antibiotic utilization (Days of Therapy per 1,000 Patient-Days) | 258.7 | 342.1 | <0.001 |  |
|  |  |  |  |  |  |  |  |  | ID consultations (per 1,000 PD) | 21.5 | 15.4 | 0.001 |  |
|  |  |  |  |  |  |  |  |  | Annual expenditures saved | $142,629.83 |  |  |  |
| Wilson^27^; 2019 | prospective quasi-experimental study | USA | Site A (n=27 acute care beds) | NR | NR | Weekly 1-hour telehealth sessions (videoconference) to discuss concerns related to infections and  antimicrobial use among patients at the intervention site | 12 months | NR | Days of therapy/1,000 days of care, in acute care | 911.5 (885.5–938.0) | 984.7 (959.0–1010.8) | <0.001 | a |
|  |  |  |  |  |  |  |  |  | Days of therapy/1,000 days of care, in long-term care | 72.1 (69.8–74.4) | 89.7 (87.1–92.3) | <0.001 | b |
|  |  |  |  |  |  |  |  |  | Antibiotic spectrum index mean (95%CI), in acute care | 8.42 (8.26–8.57) | 8.76 (8.62–8.90) | 0.001 |  |
|  |  |  |  |  |  |  |  |  | Antibiotic spectrum index mean (95%CI), in long-term care | 5.84 (5.74–5.93) | 6.44 (6.34– 6.55) | <0.001 |  |
|  |  |  |  |  |  |  |  |  | Length of therapy mean (95%CI), in acute care | 3.55 (3.40–3.70) | 4.01 (3.84–4.17) | <0.001 |  |
|  |  |  |  |  |  |  |  |  | Length of therapy mean (95%CI), in long-term care | 6.46 (5.22–7.70) | 7.11 (6.50–7.71) | 0.359 |  |
|  |  |  | Site B (n=10 acute care beds) | NR | NR | Weekly 1-hour telehealth sessions (videoconference) to discuss concerns related to infections and  antimicrobial use among patients at the intervention site | 12 months | NR | Days of therapy/1,000 days of care, in acute care | 453.3 (424.0–484.1) | 577.3 (542.1–614.1) | <0.001 | c |
|  |  |  |  |  |  |  |  |  | Days of therapy/1,000 days of care, in long-term care | 69.0 (66.6–71.3) | 81.9 (79.3–84.5) | <0.001 | d |
|  |  |  |  |  |  |  |  |  | Antibiotic spectrum index mean (95%CI), in acute care | 6.59 (6.35–6.82) | 6.73 (6.48–6.99) | 0.400 |  |
|  |  |  |  |  |  |  |  |  | Antibiotic spectrum index mean (95%CI), in long-term care | 5.45 (5.35–5.54) | 5.25 (5.17–5.33) | 0.001 |  |
|  |  |  |  |  |  |  |  |  | Length of therapy mean (95%CI), in acute care | 3.10 (2.83–3.38) | 3.15 (2.94–3.36) | 0.803 |  |
|  |  |  |  |  |  |  |  |  | Length of therapy mean (95%CI), long term care | 6.56 (5.91–7.21) | 7.23 (5.58–8.88) | 0.457 |  |
| Avent^34^; 2021 | Retrospective cohort | Queensland, Australia | Four rural hospital and health sevice facilities in Queensland with a total of 8 to 23 inpatient beds per hospital | NR | NR | Telephone hotline for consultation with antimicrobial stewardship team; monthly education of staff using telehealth; weekly ward rounds of antimicrobial stewardship conducted using telehealth | 12 months | January 2017 - December 2018 | Adherence to guidelines | 54.1% (48.7 - 59.5)  aOR:  2.44, 95% CI: 1.70-3.51 | 33.7% (27.0 - 40.4) | <0.001 |  |
|  |  |  |  |  |  |  |  |  | Appropriateness of prescribing | 67.5% (48,7 - 59.5)  aOR  2.48, 95% CI: 1.70-3.61 | 49% (42.2 - 55.9) | <0.001 |  |
| Beulac^35^; 2016 | Retrospective cohort | USA | Long term acute care hospital with 212 beds | Long term acute care patients (inpatient care >25 days) | 68 (34) | Antimicrobial recommendations provided through e-mail by a team of infectious disease physicians and trained pharmacists | 36 months | April 2011 - March 2014 | Total antibiotics usage (defined daily doses/1000 patient-days) | 259.42 | 266 | 0.01 |  |
|  |  |  |  |  |  |  |  |  |  | −6.58 (−11.48 - −1.67) | |  |  |
| Charani^36^; 2017 | Retrospective cohort | London, UK | Three main hospitals in London, with a total of 1300 beds | NR | NR | Antibiotics prescribing policy available through an application | 36 months | August 2011 - August 2014 | Increased compliance with policy in surgery | 6.48% (-1.25 - 14.20) | 0.79% (-0.78 - 2.36) | <0.05 |  |
|  |  |  |  |  |  |  |  |  | Increased compliance with policy in medicine | 6.63% (0.15 - 13.10) | 0.21% (-1.29 - 1.71) | >0.05 |  |
| Klatt^9^; 2021 | Quasi- experimental study | USA | Preintervention period (n=395)  Intervention (n=326)  Postintervention (n=316) | Patients with pneumonia, urinary tract, or skin and soft tissue infection | 69.6 (19.4) | Antimicrobial stewardship  services including prospective audit  and feedback, guideline and order  set management, and staff education delivered via mobile application | 7 months | June 2013-December 2015 | **Reduction of antibiotic use**    Imipenem  Levofloxacin  Piperacilin/ tazobactam  Vancomycin | Decrease 62.7%  Decrease 19.7%  Decrease 7.1%  Decrease 18.0% | | <.001  <.001  0.12  <.001 |  |
|  |  |  |  |  |  |  |  |  | Imipenem use (DOT/1,000 DP) | 31 | 83 | <.001 |  |
|  |  |  |  |  |  |  |  |  | Levofloxacin use (DOT/1,000 DP) | 99 | 123 | <.001 |  |
|  |  |  |  |  |  |  |  |  | Piperacilin/tazobactam use (DOT/1,000 DP) | 142 | 153 | <.001x |  |
|  |  |  |  |  |  |  |  |  | Vancomycin use (DOT/1,000 DP) | 85 | 104 | <.001 |  |
|  |  |  |  |  |  |  |  |  | Mean length of stay | 4.2 [2.6] days | 4.6 [2.8] days | <.049 |  |
|  |  |  |  |  |  |  |  |  | In hospital mortality | 12.6% | 9.4% | N/A |  |
|  |  |  |  |  |  |  |  |  | 30-day mortality rate | 11.4% | 9.5% | .47 |  |
| Meredith^37^; 2021 | Retrospective cohort study | USA | 10 hospitals with number of prescriptions:  Intervention group  (n=576)  Control group  (n=162) | Hospitalized adult patients with *Staphylococcus*  *aureus* bacteremia | 58 (45–70) | Antimicrobial Stewardship Program for *Staphylococcus aureus* bacteremia bundle of guidelines was delivered using videoconference method | 16 months | September 2016-December 2017 | Adherence to guidelines | 144 (88.9%) | 495 (85.9%) | .33 |  |
|  |  |  |  |  |  |  |  |  | 30-day mortality | 27 | 64 | .08 |  |
|  |  |  |  |  |  |  |  |  | Hospital mortality | 14 | 43 | .62 |  |
| Ray^38^; 2021 | Retrospective cohort study | USA | Pediatric primary care | Patients with acute respiratory tract infection  Intervention group (n=3,003)  Control group (n=5,329) | NR | Interactive dashboard including guideline-concordant antibiotic management  and antibiotic prescriptions for in-person and  telemedicine ARTI visits delivered using videoconference | 6 months | April-September 2020 | Guideline- concordant  antibiotics | 92.5%  (2779/3003) | 90.7%  (4832/5329) | .004 |  |
| Yam^15^, 2012 | Observational longitudinal | USA | Community rural hospital with 141 licensed beds | N/A | N/A | Antimicrobial stewardship program with the involvement of a remotely located ID physician specialist for case reviews via teleconferencing  patients  (n = 311) | 13 months | May 2010- June 2011 | Number of intervention after review of antimicrobial therapy by the clinical pharmacist | 6.8/week (postintervention) | 2.1/week | NR |  |
|  |  |  |  |  |  |  |  |  | Rate of antimicrobial streamlining | 96% | 44% | NR |  |
|  |  |  |  |  |  |  |  |  | The percentage agreement between pharmacist and ID physician recommendations | 86% | NR | NR |  |
|  |  |  |  |  |  |  |  |  | Cost savings associated with AMS activities | $6,583.52 (decrease 51.3%) | $13,521 | NR |  |
|  |  |  |  |  |  |  |  |  | Clostridium difficile infection rates before and after program implementation. | 3.1 cases/10.000 patients-days  (decrease 62.2%) | 8.2 cases/10.000 patients-days | NR |  |
| Du Yan^39^, 2021 | Randomized Controlled Trial | USA | Telemedicine direct-to-patient video visit | All Clinicians Employed at Practice (n=45)  patient visits (n=55364) ​​ | NR | Intervention = Education via 1-slide presentation and course + Individualized feedback via online dashboards summarizing prescription rates  (n=22)  Control=  Education Only (n=23) | 10  months | January 1 2018-November 30 2018 | Prescription adjusted odds ratio of prescription rates for Upper Respiratory Infection (URI) | 0.60 [0.47-0.77] | | NR | a |
|  |  |  |  |  |  |  |  |  | Adjusted odds ratio of prescription rates for Bronchitis | 0.42 [0.32-0.55] | | NR | b |
|  |  |  |  |  |  |  |  |  | Adjusted odds ratio of prescription rates for Sinusitis | 1.05 [0.91-1.21] | | NR | c |
|  |  |  |  |  |  |  |  |  | Adjusted odds ratio of prescription rates for Pharyngitis | 0.91 [0.76-1.09] | | NR | d |
| Wasylyshyn^40^, 2022 | Cohort study | USA | E-visits responded by an advanced practice provider | Patients requesting care for cough, flu, or sinus symptoms | 49 (preintervention)  44 (postintervention) | Change of questionnaire to assess guideline-concordant diagnoses, electronic medical records enhancement, support tools to give nudges towards guideline-concordant prescription, guideline development by antimicrobial stewardship committee, real-time physicians reviewing prescription rates and providing audits on guideline concordance  Preintervention Period: Jan 1 to Dec 31, 2018  (n= 972)  Postintervention Period: Jun 1, 2019,  to Sep 30, 2020  (n= 3,562) | 32 months | January 2018-September 2020 | **Antibiotics prescribed, no, (%)** | **Postintervention** | **Preintervention** |  |  |
|  |  |  |  |  |  |  |  |  | Antibiotics prescribed for sinusitis | 921 (56.4% of sinusitis visits) | 342 (69.9% of sinusitis visits) | <.001 | a |
|  |  |  |  |  |  |  |  |  | Antibiotics prescribed for flu | 107 (5.5% of flu visits) | 71 (15.6% of flu visits) | <.001 | b |
|  |  |  |  |  |  |  |  |  | **Antibiotic choice** |  | |  |  |
|  |  |  |  |  |  |  |  |  | Amoxicillin-clavulanate | 680 (66.1% of visits with  prescriptions) | 159 (37.9% of visits with prescriptions) | <.001 |  |
|  |  |  |  |  |  |  |  |  | Doxycycline | 234 (22.7% of visits with  prescriptions) | 58 (13.8% of visits with prescriptions) | <.001 |  |
|  |  |  |  |  |  |  |  |  | Azithromycin | 54 (5.3% of visits with  prescriptions) | 73 (17.3% of visits with prescriptions) | <.001 |  |
|  |  |  |  |  |  |  |  |  | Other | 60 (5.8% of visits with  prescriptions) | 203 (48.3% of visits with prescriptions) | <.001 |  |
|  |  |  |  |  |  |  |  |  | **Median duration, d** | 5 | 10 | <.001 |  |
|  |  |  |  |  |  |  |  |  | Median duration if azithromycin prescriptions excluded, d | 7 | 10 | <.001 |  |
|  |  |  |  |  |  |  |  |  | **Follow-up visit within 14 days when antibiotics were**  **prescribed** | 104 (2.9% of all visits) | 44 (4.5% of all visits) | 0.02 |  |
| Ceradini^22^, 2017 | Cohort study | Italy | Pediatric cardiology clinical center, part of Taormina hospital housing 220 beds | Pediatric cardiology patients, including children in critical situations or requiring long-period hospital support | Median age preintervention :1.7(0.2-7.4)  Postintervention:  2.1 (0..2-9.5) | Biweekly discussion of clinical cases and review of antiobiotics using telepresence online meetings or real-time video conferencing  before intervention admissions (n=683)  post intervention admission (n=531) | 12 months | 1 January 2014 -1 March 2016 | average stay in pediatric ICU (days) | 6.1±12.9  (postintervention) | 6.2 ±10.8 | 0.92 |  |
|  |  |  |  |  |  |  |  |  | average hospital stay (days) | 8.4±11.7  (postintervention) | 8.4±11.9 | NR |  |
|  |  |  |  |  |  |  |  |  | ICU infection rate per 1000 days people | 6.05 (postintervention) | 9.5 | 0.23 |  |
|  |  |  |  |  |  |  |  |  | Multi drug resistant isolation rate | 79  (postintervention) | 104 | 0.01 |  |
|  |  |  |  |  |  |  |  |  | Average weight diagnostic related group | 2  (postintervention) | 2.3 | NR |  |

**Appendix 2.** Risk of Bias Assessment with ROBINS-I

|  | **Signalling questions** | **Wilson; 2019** | **Klatt; 2021** | **Vento; 2021** | **Shively; 2019** | **Response options** |
| --- | --- | --- | --- | --- | --- | --- |
| **Bias due to confounding** | | | | | | |
|  | 1.1 Is there potential for confounding of the effect of intervention in this study?  **If N/PN to 1.1:** the study can be considered to be at low risk of bias due to confounding and no further signalling questions need be considered | N | N | PN | PN | Y / PY / PN / N |
|  | **If Y/PY to 1.1**: determine whether there is a need to assess time-varying confounding: |  |  |  |  |  |
|  | 1.2. Was the analysis based on splitting participants’ follow up time according to intervention received?  **If N/PN**, answer questions relating to baseline confounding (1.4 to 1.6)  **If Y/PY**, go to question 1.3. | PY | Y | PY | Y | NA / Y / PY / PN / N / NI |
|  | 1.3. Were intervention discontinuations or switches likely to be related to factors that are prognostic for the outcome?  **If N/PN**, answer questions relating to baseline confounding (1.4 to 1.6)  **If Y/PY**, answer questions relating to both baseline and time-varying confounding (1.7 and 1.8) | PN | N | PN | PN | NA / Y / PY / PN / N / NI |

|  | **Questions relating to baseline confounding only** | | | | | |
| --- | --- | --- | --- | --- | --- | --- |
|  | 1.4. Did the authors use an appropriate analysis method that controlled for all the important confounding domains? | NI | Y | PN | PY | NA / Y / PY / PN / N / NI |
|  | 1.5. **If Y/PY to 1.4**: Were confounding domains that were controlled for measured validly and reliably by the variables available in this study? | NI | Y | NA | PY | NA / Y / PY / PN / N / NI |
|  | 1.6. Did the authors control for any post-intervention variables that could have been affected by the intervention? | PN | N | N | N | NA / Y / PY / PN / N / NI |
|  | **Questions relating to baseline and time-varying confounding** | | | | |  |
|  | 1.7. Did the authors use an appropriate analysis method that controlled for all the important confounding domains and for time-varying confounding? |  | Y | PN | PY | NA / Y / PY / PN / N / NI |
|  | 1.8. **If Y/PY to 1.7**: Were confounding domains that were controlled for measured validly and reliably by the variables available in this study? |  | Y | NA | PY | NA / Y / PY / PN / N / NI |
|  | **Risk of bias judgement** | Low | Low | Moderate | Low | Low / Moderate / Serious / Critical / NI |

| **Bias in selection of participants into the study** | | | | | | |
| --- | --- | --- | --- | --- | --- | --- |
|  | 2.1. Was selection of participants into the study (or into the analysis) based on participant characteristics observed after the start of intervention?  **If N/PN to 2.1:** go to 2.4 | N | N | N | PN | Y / PY / PN / N / NI |
|  | 2.2. **If Y/PY to 2.1**: Were the post-intervention variables that influenced selection likely to be associated with intervention?  2.3 **If Y/PY to 2.2**: Were the post-intervention variables that influenced selection likely to be influenced by the outcome or a cause of the outcome? | NA | NA | NA | NA | NA / Y / PY / PN / N / NI  NA / Y / PY / PN / N / NI |
|  | 2.4. Do start of follow-up and start of intervention coincide for most participants? | PY | Y | PN | PY | Y / PY / PN / N / NI |
|  | 2.5. **If Y/PY to 2.2 and 2.3, or N/PN to 2.4**: Were adjustment techniques used that are likely to correct for the presence of selection biases? | NA | NA | PN | N/A | NA / Y / PY / PN / N / NI |
|  | **Risk of bias judgement** | Low | Low | Moderate | Low | Low / Moderate / Serious / Critical / NI |

| **Bias in classification of interventions** | | | | | | |
| --- | --- | --- | --- | --- | --- | --- |
|  | 3.1 Were intervention groups clearly defined? | PY | Y | Y | Y | Y / PY / PN / N / NI |
|  | 3.2 Was the information used to define intervention groups recorded at the start of the intervention? | PY | Y | Y | Y | Y / PY / PN / N / NI |
|  | 3.3 Could classification of intervention status have been affected by knowledge of the outcome or risk of the outcome? | PN | N | PN | PN | Y / PY / PN / N / NI |
|  | **Risk of bias judgement** | Low | Low | Low | Low | Low / Moderate / Serious / Critical / NI |

| **Bias due to deviations from intended interventions** | | | | | | |
| --- | --- | --- | --- | --- | --- | --- |
|  | **If your aim for this study is to assess the effect of assignment to intervention, answer questions 4.1 and 4.2** | | | | |  |
|  | 4.1. Were there deviations from the intended intervention beyond what would be expected in usual practice? | N | N | PN | PN | Y / PY / PN / N / NI |
|  | 4.2. **If Y/PY to 4.1**: Were these deviations from intended intervention unbalanced between groups *and* likely to have affected the outcome? | N/A | N | N/A | N/A | NA / Y / PY / PN / N / NI |
|  | **If your aim for this study is to assess the effect of starting and adhering to intervention, answer questions 4.3 to 4.6** | | | | |  |
|  | 4.3. Were important co-interventions balanced across intervention groups? | Y | Y | PY | PY | Y / PY / PN / N / NI |
|  | 4.4. Was the intervention implemented successfully for most participants? | Y | Y | Y | Y | Y / PY / PN / N / NI |
|  | 4.5. Did study participants adhere to the assigned intervention regimen? | PY | Y | Y | Y | Y / PY / PN / N / NI |
|  | 4.6. **If N/PN to 4.3, 4.4 or 4.5**: Was an appropriate analysis used to estimate the effect of starting and adhering to the intervention? | NA | NA | NA | NA | NA / Y / PY / PN / N / NI |
|  | **Risk of bias judgement** | Low | Low | Low | Low | Low / Moderate / Serious / Critical / NI |

| **Bias due to missing data** | | | | | | |
| --- | --- | --- | --- | --- | --- | --- |
|  | 5.1 Were outcome data available for all, or nearly all, participants? | PY | Y | PY | PY | Y / PY / PN / N / NI |
|  | 5.2 Were participants excluded due to missing data on intervention status? | PN | N | N | PN | Y / PY / PN / N / NI |
|  | 5.3 Were participants excluded due to missing data on other variables needed for the analysis? | PN | N | N | PN | Y / PY / PN / N / NI |
|  | 5.4 **If PN/N to 5.1, or Y/PY to 5.2 or 5.3**: Are the proportion of participants and reasons for missing data similar across interventions? | NA | NA | NA | NA | NA / Y / PY / PN / N / NI |
|  | 5.5 **If PN/N to 5.1, or Y/PY to 5.2 or 5.3**: Is there evidence that results were robust to the presence of missing data? | NA | NA | NA | NA | NA / Y / PY / PN / N / NI |
|  | **Risk of bias judgment** | Low | Low | Low | Low | Low / Moderate / Serious / Critical / NI |

| **Bias in measurement of outcomes** | | | | | | |
| --- | --- | --- | --- | --- | --- | --- |
|  | 6.1 Could the outcome measure have been influenced by knowledge of the intervention received? | PN | N | N | PY | Y / PY / PN / N / NI |
|  | 6.2 Were outcome assessors aware of the intervention received by study participants? | NI | N | PY | PN | Y / PY / PN / N / NI |
|  | 6.3 Were the methods of outcome assessment comparable across intervention groups? | PY | Y | Y | Y | Y / PY / PN / N / NI |
|  | 6.4 Were any systematic errors in measurement of the outcome related to intervention received? | PN | N | PN | PN | Y / PY / PN / N / NI |
|  | **Risk of bias judgement** | Moderate | Low | Moderate | Moderate | Low / Moderate / Serious / Critical / NI |

| **Bias in selection of the reported result** | | | | | | |
| --- | --- | --- | --- | --- | --- | --- |
|  | Is the reported effect estimate likely to be selected, on the basis of the results, from... | PN | N | PN | PN | Y / PY / PN / N / NI |
|  | 7.1. ... multiple outcome *measurements* within the outcome domain? |  |  |  |  |  |
|  | 7.2 ... multiple *analyses* of the intervention-outcome relationship? | PN | N | PN | PN | Y / PY / PN / N / NI |
|  | 7.3 ... different *subgroups*? | PN | N | N | N | Y / PY / PN / N / NI |
|  | **Risk of bias judgement** | Low | Low | Low | Low | Low / Moderate / Serious / Critical / NI |

| **Overall bias** | | | | | | |
| --- | --- | --- | --- | --- | --- | --- |
|  | **Risk of bias judgement** | Low | Low | Moderate | Low | Low / Moderate / Serious / Critical / NI |

**Appendix 3.** Risk of Bias Assessment with using Cochrane Risk of Bias Tool 2.0 for Randomized Studies

|  | **Signalling questions** | **Response options** | **Du Yan; 2021** |
| --- | --- | --- | --- |
|  | **Bias arising from the randomization process** | | |
|  | 1.1 Was the allocation sequence random? | Y / PY / PN / N / NI | Y |
|  | 1.2 Was the allocation sequence concealed until participants were enrolled and assigned to interventions? | Y / PY / PN / N / NI | Y |
|  | 1.3 Did baseline differences between intervention groups suggest a problem with the randomization process? | Y / PY / PN / N / NI | N |
|  | Risk of bias judgement | **- / + / ?** | **Low risk** |

|  | **Domain 2: Risk of bias due to deviations from the intended interventions (effect of assignment to intervention)** | | |
| --- | --- | --- | --- |
|  | 2.1. Were participants aware of their assigned intervention during the trial? | Y / PY / PN / N / NI | PY |
|  | 2.2. Were carers and people delivering the interventions aware of participants' assigned intervention during the trial? | Y / PY / PN / N / NI | PY |
|  | 2.3. If Y/PY/NI to 2.1 or 2.2: Were there deviations from the intended intervention that arose because of the experimental context? | NA / Y / PY / PN / N / NI | N |
|  | 2.4. If Y/PY to 2.3: Were these deviations from intended intervention balanced between groups? | NA / Y / PY / PN / N / NI | NA |
|  | 2.5 If N/PN/NI to 2.4: Were these deviations likely to have affected the outcome? | NA / Y / PY / PN / N / NI | NA |
|  | 2.6 Was an appropriate analysis used to estimate the effect of assignment to intervention? | Y / PY / PN / N / NI | PY |
|  | 2.7 If N/PN/NI to 2.6: Was there potential for a substantial impact (on the result) of the failure to analyse participants in the group to which they were randomized? | NA / Y / PY / PN / N / NI | NA |
|  | **Risk of bias judgement** | **- / + / ?** | **Some concerns** |

|  | **Domain 2: Risk of bias due to deviations from the intended interventions (effect of adhering to intervention)** | | |
| --- | --- | --- | --- |
|  | 2.1. Were participants aware of their assigned intervention during the trial? | Y / PY / PN / N / NI | PY |
|  | 2.2. Were carers and people delivering the interventions aware of participants' assigned intervention during the trial? | Y / PY / PN / N / NI | PY |
|  | 2.3. If Y/PY/NI to 2.1 or 2.2: Were important co-interventions balanced across intervention groups? | NA / Y / PY / PN / N / NI | PY |
|  | 2.4. Were there failures in implementing the intervention that could have affected the outcome? | Y / PY / PN / N / NI | PN |
|  | 2.5. Was there non-adherence to the assigned intervention regimen that could have affected participants’ outcomes? | Y / PY / PN / N / NI | N |
|  | 2.6. If N/PN/NI to 2.3 or 2.5 or Y/PY/NI to 2.4: Was an appropriate analysis used to estimate the effect of adhering to the intervention? | NA / Y / PY / PN / N / NI | NA |
|  | **Risk of bias judgement** | **- / + / ?** | **Low risk** |

|  | **Bias due to missing outcome data** | | |
| --- | --- | --- | --- |
|  | 3.1 Were data for this outcome available for all, or nearly all, participants randomized? | Y / PY / PN / N / NI | Y |
|  | 3.2 If N/PN/NI to 3.1: Is there evidence that the result was not biased by missing outcome data? | NA / Y / PY / PN / N | NA |
|  | 3.3 If N/PN to 3.2: Could missingness in the outcome depend on its true value? | NA / Y / PY / PN / N / NI | NA |
|  | 3.4 If Y/PY/NI to 3.3: Is it likely that missingness in the outcome depended on its true value? | NA / Y / PY / PN / N / NI | NA |
|  | **Risk of bias judgement** | **- / + / ?** | **Low risk** |

|  | **Bias in measurement of the outcome** | | |
| --- | --- | --- | --- |
|  | 4.1 Was the method of measuring the outcome inappropriate? | Y / PY / PN / N / NI | N |
|  | 4.2 Could measurement or ascertainment of the outcome have differed between intervention groups? | Y / PY / PN / N / NI | PN |
|  | 4.3 If N/PN/NI to 4.1 and 4.2: Were outcome assessors aware of the intervention received by study participants? | Y / PY / PN / N / NI | N |
|  | 4.4 If Y/PY/NI to 4.3: Could assessment of the outcome have been influenced by knowledge of intervention received? | NA / Y / PY / PN / N / NI | NA |
|  | 4.5 If Y/PY/NI to 4.4: Is it likely that assessment of the outcome was influenced by knowledge of intervention received? | NA / Y / PY / PN / N / NI | NA |
|  | **Risk of bias judgement** | **- / + / ?** | **Low risk** |

|  | **Bias in selection of the reported result** | | |
| --- | --- | --- | --- |
|  | 5.1 Were the data that produced this result analysed in accordance with a pre-specified analysis plan that was finalized before unblinded outcome data were available for analysis? | Y / PY / PN / N / NI | PY |
|  | Is the numerical result being assessed likely to have been selected, on the basis of the results, from... |  |  |
|  | 5.2. ... multiple outcome measurements (e.g. scales, definitions, time points) within the outcome domain? | Y / PY / PN / N / NI | N |
|  | 5.3 ... multiple analyses of the data? | Y / PY / PN / N / NI | PN |
|  | **Risk of bias judgement** | **- / + / ?** | **Low risk** |

|  | **Overall bias** | | |
| --- | --- | --- | --- |
|  | **Risk of bias judgement** | **- / + / ?** | **Some concerns** |

**Appendix 4.** Funnel Plot Analysis for Heterogeneity (A) For adherence to guidelines (B) For antimicrobial prescription (C) For days of therapy (D) For mortality rate

A**
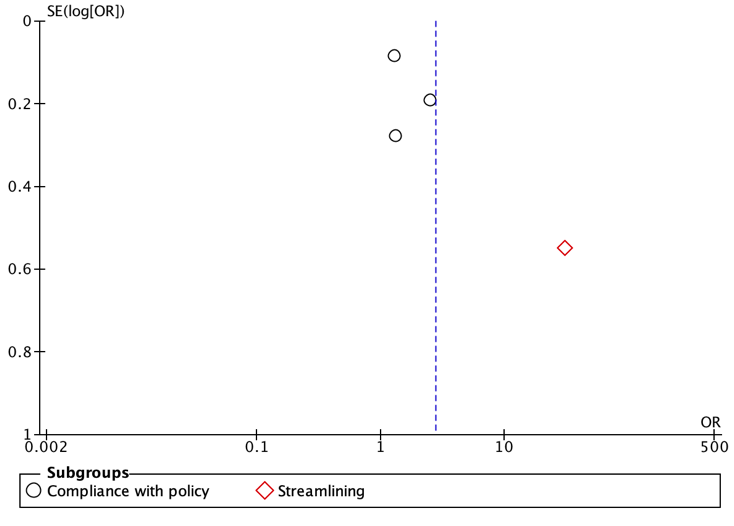
**

**B
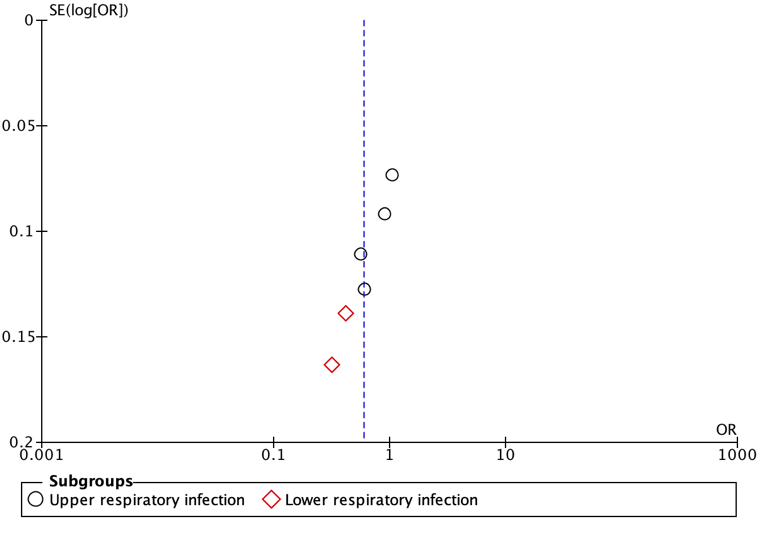
**

**C**
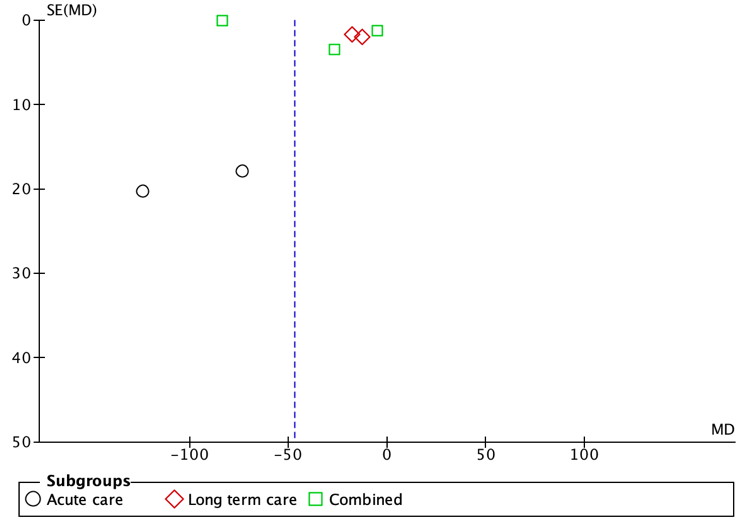


**D**
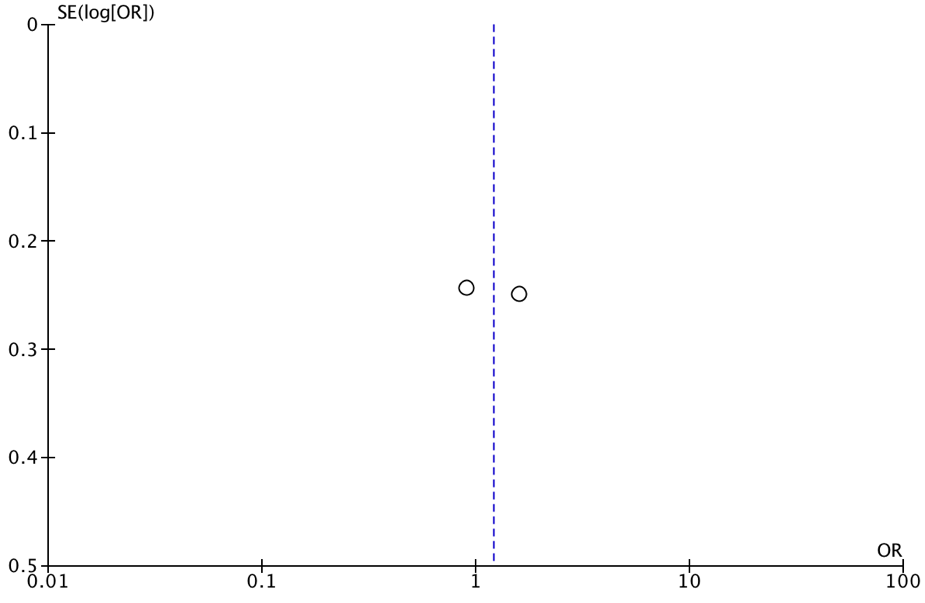


**Appendix 5.** Details of Statistical Calculations for Days of Therapy


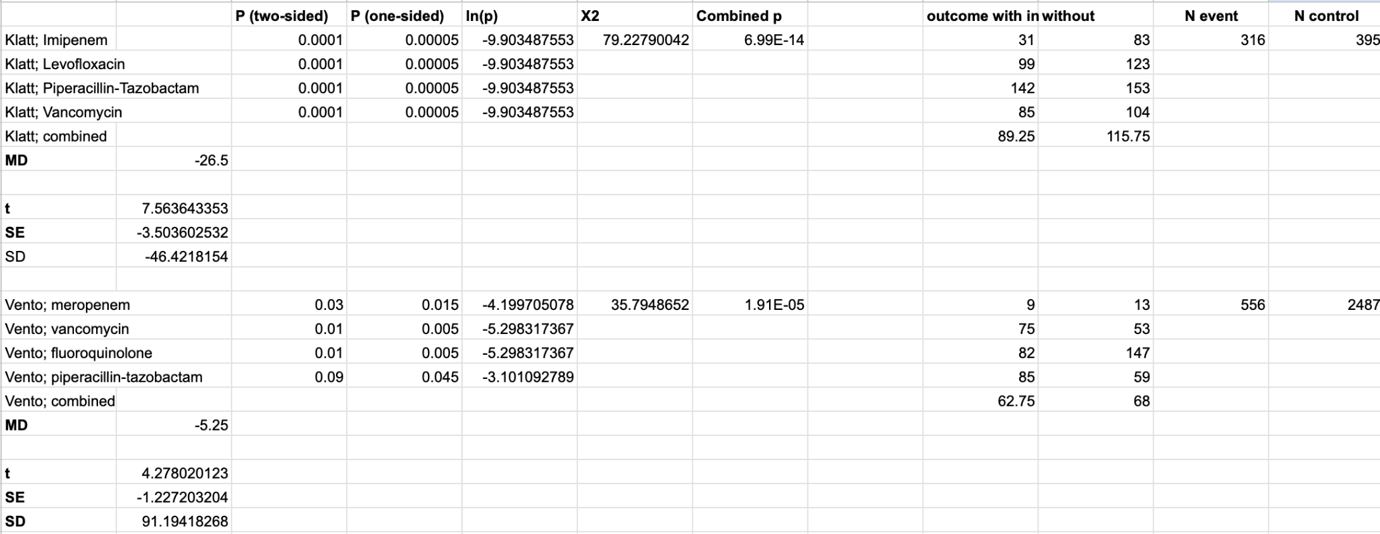

Supplement: sj-docx-1-jtt-10.1177_1357633X231204919 - Supplemental material for Efficacy of telemedicine-based antimicrobial stewardship program to combat antimicrobial resistance: A systematic review and meta-analysis [file sj-docx-1-jtt-10.1177_1357633X231204919.docx]
